# Overview of Adult Sarcoma Burden and Clinical Pathways in Brazil

**DOI:** 10.1200/GO.21.00387

**Published:** 2022-03-01

**Authors:** Bruna Bianca Lopes David, Celso Abdon Mello, Luiz Claudio Santos Thuler, Andreia Cristina de Melo

**Affiliations:** ^1^ Division of Clinical Research and Technological Development, Brazilian National Cancer Institute, Rio de Janeiro, Brazil; ^2^ Department of Medical Oncology, A.C Camargo Cancer Center, São Paulo, Brazil

## Abstract

Sarcomas are a heterogeneous group of cancers classified as rare cancers and are often poorly characterized. In Brazil, little is known about the adult sarcoma burden and patients' clinical pathways.

**MATERIALS AND METHODS:**

We analyzed data from the Brazilian Hospital–Based Cancer Registries System, which encompasses the entire country. The histologic criteria included sarcomas according to the International Classification of Diseases for Oncology, 3rd edition. All cases were histology-based**.** No central pathology review was performed. Patients < 18 years old were excluded. The variables were analyzed according to the center type, hospital patient volume (> 70 patients/year for 3 consecutive years), and geographical region. The results were based on valid data, and the missing values were reported.

**RESULTS:**

From 2000 to 2017, a total of 312 units and 49,878 cases were identified. Missing data proportion was stable. Soft tissue sarcomas were predominant, followed by bone sarcomas and gastrointestinal stromal tumors. The Southeast concentrated on the largest number of patients (51%), of high-complexity centers (CACONs; 52%), and of patients treated at CACONs (56.9%). In all regions, the majority of patients had localized disease at diagnosis. The proportion of patients starting their treatment within 60 days from diagnosis at CACON was 59.3% and 62.3% at others. Ten hospitals achieved the established threshold for high-volume center, of which seven were CACON.

**CONCLUSION:**

This article highlights the need for further research on the profile of patients with sarcoma in Brazil and the importance of providing them a more effective diagnostic and therapeutic approach. This initiative is critical not just for planning treatment strategies but also to allocate medical resources and to improve quality of care and sarcoma patients outcomes.

## INTRODUCTION

Sarcomas are a heterogeneous group of cancers arising from soft tissues and bone. They are classified as rare cancers, as their annual incidence is < 6 per 100,000 people, accounting for < 2% of all adult solid tumors.^1-3^ Bone sarcoma (BS) and soft tissue sarcoma (STS) include about 100 different pathologic entities, as described in the 2020 WHO Classification of Tumours—Soft Tissue and Bone Tumours, many of which are ultrarare (incidence < 1 per million).^4,5^ Because of their rarity, sarcomas are often poorly characterized with regard to their epidemiology, biology, natural history, prognostic and predictive factors, and sensitivity to standard treatment, which pose challenges for diagnosis and clinical decision making.

**CONTEXT**

**Key Objective**
Is quality information on rare cancers luxury or necessity in developing countries?
**Knowledge Generated**
In Brazil, as in many other low- and middle-income countries, data about sarcomas are scarce at a national level. Regarding access to care, patients with sarcoma face significant hurdles, with as many as 40% starting their treatment after 60 days of diagnosis. Several obstacles, such as access barriers, the absence of a specific clinical flow for those patients, and inadequate facilities and personnel, may contribute to worse indicators.
**Relevance**
Comprehensive cancer registry and data quality are critical to map rare cancers. The picture framed here generates data to better understand the reality and clinical pathways of adult patients with sarcomas in Brazil and where there is a room for improvement to better direct efforts and allocate medical resources.

The broad consensus suggests referring and managing patients with sarcoma in reference centers with a high yearly volume of new cases. Collaborative networks with very specific expertise and a multidisciplinary approach also improve patient outcomes^6-11^ and are fundamental to promote clinical and translational research.^5,12^ Cooperation should also be a motto when it comes to data registration. In Europe, the RARECARE and thereafter RARECAREnet projects were funded by the European Commission to provide a definition and, more importantly, to identify the burden in terms of incidence, prevalence, and survival of low-incidence cancers, including sarcomas.^3^ These projects combined data from more than 90 population-based cancer registries and were instrumental to motivate actions around the world to improve health services organization and research methodology in rare cancers.^13^

In Brazil, as in many other low- and middle-income countries, information about sarcomas at the national level is poor. Every 3 years, the Brazilian National Cancer Institute (INCA) reports incidence estimates on the basis of data from 27 Population-Based Cancer Registries, integrating information from 321 Hospital Cancer databases. Sarcoma data are computed as other topographies in a mixed group with other low-incidence cancers.^14^ In practice, specific data about rare cancers come from hospital-based registries. This modality is useful for monitoring and evaluating the quality of care but has some limitations: institution databases are not homogenous, the population covered is limited to the hospital area, and there is the possibility of double registry for the same case, if a patient migrates or has more than one center involved in the cancer care pathway. The creation of population-based registries should be encouraged to better characterize each disease profile.

In addition to its scarce registry system, Brazil is a continental country with diverse and highly mixed populations.^15^ Geographically, it is composed of 26 states and the Federal District, divided into five regions, the North (N), Northeast (NE), South (S), Southeast (SE), and Middle-West (MW). These regions are distinct in terms of the development index, in which the N/NE are the poorest (Human Development Index [HDI] = 0.667 and 0.663, respectively) and the S/SE/MW are the richest (HDI = 0.754, 0.766, and 0.757, respectively), resulting in social, economic, and health care inequalities.^16^ The disparities also affect the Brazilian health organization. The system is marked by a dualism, whereby 20% of the population has access to private/supplemental health and the remaining 80% is covered by a Unified Health System (Sistema Único de Saúde [SUS]). SUS faces budgetary, governance, and organizational limitations, which affects its sustainability and capability to provide adequate health care. In this context, the management of rare conditions, such as sarcomas, has been deeply affected.

In view of the above scenario, this study aims to estimate the sarcoma burden in Brazil and to provide information about these patients' clinical pathways using the national hospital–based data. The data generated here could be useful for other low- and middle-income countries around the world, especially in Latin America, to cooperate and better structure their health care systems, improving sarcoma care and local policy decisions.

## MATERIALS AND METHODS

The current study analyzed data from the Brazilian Hospital–Based Cancer Registries System^17^ from the five geographical regions: NE, N, S, SE, and MW, encompassing the whole territory, with differences in HDI and cancer policies. A total of 312 hospital units in the 26 states and the Federal District were included to estimate sarcoma characteristics from 2000 to 2017 and to describe the initial clinical path of these patients across the country. The population covered could not be calculated because of years of information disruption by some centers and the unavailability of health migration data. According to the International Classification of Diseases for Oncology, Third Edition (ICD-O-3),^18^ the histologic criteria included STS (all sites except C40.0-C41.9), BS (only C40.0-C41.9), and gastrointestinal stromal sarcomas (GIST; any site). All cases were histology-based. No central pathology review was conducted, and patients under age 18 years were excluded.

Patients were monitored until the end of the first course of a sarcoma-directed treatment, considering the most frequent modalities: surgery, chemotherapy, and/or radiotherapy. The following variables were collected: date of diagnosis, age at diagnosis, sex, ethnicity/skin color, level of education (according to the Brazilian Institute of Geography and Statistics [IBGE]),^15^ geographical area of the cancer center and the hospital type (High-Complexity Oncology Centers [CACON] and others), the number of high-volume centers (defined by the authors as more than 70 patients with sarcoma yearly for at least 3 consecutive years), the pathologic classification (sarcoma not otherwise specified or not), the time between diagnosis and first sarcoma-directed treatment (surgery, radiotherapy, and chemotherapy), disease status at diagnosis (localized or metastatic), first modality of therapy directed to sarcoma, and the percentage of cases whose sarcoma treatment was initiated at the institution analyzed or not (analytical case and nonanalytical case). The variables were analyzed according to the center type and geographical region. Descriptive statistics (mean and standard deviation or median and interquartile range for continuous variables and frequency for categorical variables) were used for demographic and clinical characteristics. The Kolmogorov-Smirnov test was used to check the normal distribution of the variables. The results were based on valid data, and the missing values were reported for each variable. Data were analyzed using SPSS version 21.0 (São Paulo, Brazil).

This study was approved by the Ethics in Human Research Committee of the Brazilian National Cancer Institute (Instituto Nacional de Câncer José Alencar Gomes da Silva [INCA]), Rio de Janeiro, Brazil (reference number 128/11 CAAE—0104.0.007.000-11), and is in compliance with Good Clinical Practice Guidelines.

## RESULTS

Between 2000 and 2017, a total of 49,878 cases were identified. Missing data proportion was stable all over the years across the variables analyzed. Morphological patterns of all study cases are summarized in Table 1. As demonstrated in Table 2, the distribution of cases and treatment characteristics according to the geographic region revealed the largest number of cases in SE, followed by NE, S, N, and MW. There was a predominance of female in the five regions. All regions except MW registered most of the cases in the period from 2012 to 2017. Histology subtype distribution was similar across the five regions, with a STS predominance, followed by BS and GIST. The SE and NE concentrated on the majority of patients treated at CACONs (56.9% and 57.7%, respectively). In all regions, the largest amount of cases had localized disease at diagnosis: SE 66.4%, S 60.8%, NE 66.4%, MW 63.2%, and N 61.8%. Regarding treatment, more than half of patients started the sarcoma care within 60 days of diagnosis, with MW exhibiting the highest rate (69.9%) and N exhibiting the lowest (51.8%).–Surgery was the first treatment modality across all regions, followed by chemotherapy and radiotherapy.

**TABLE 1:**
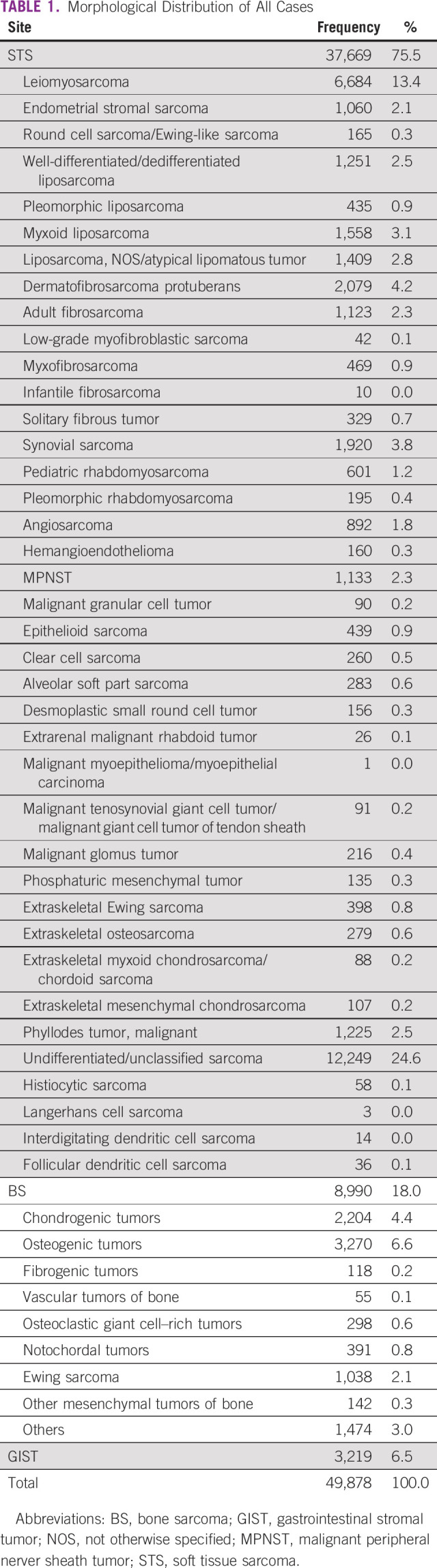
Morphological Distribution of All Cases

**TABLE 2:**
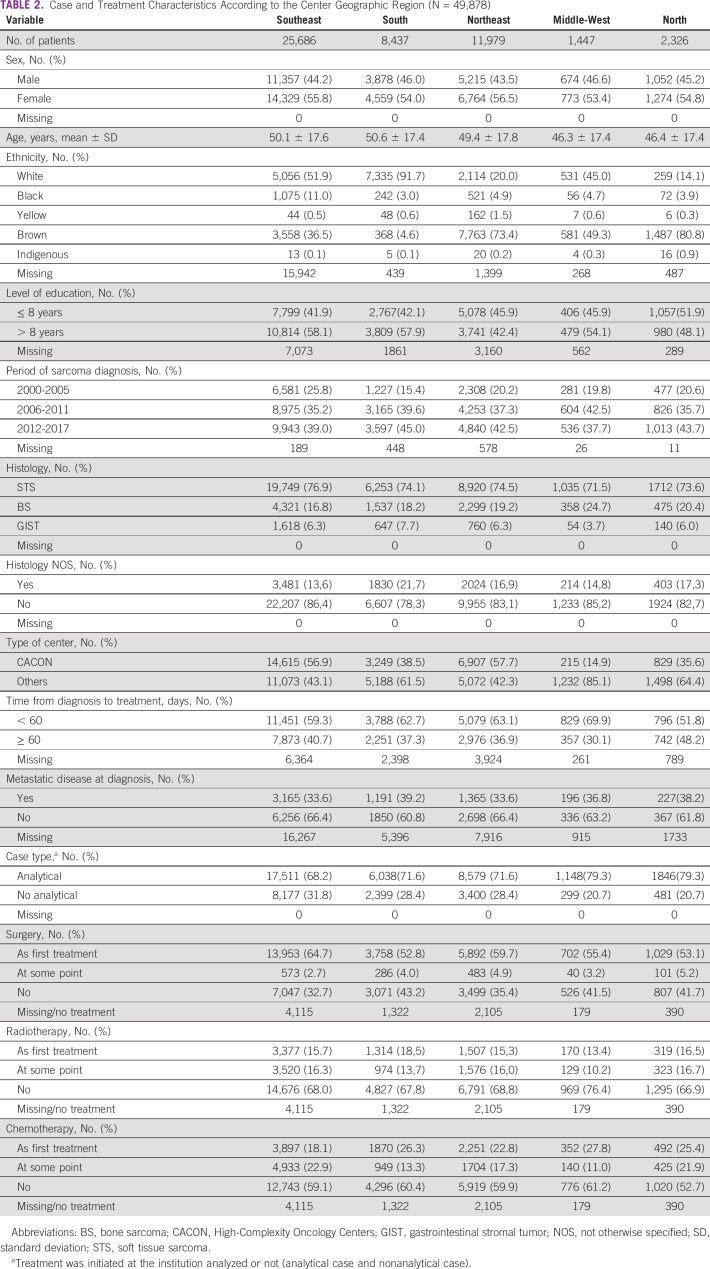
Case and Treatment Characteristics According to the Center Geographic Region (N = 49,878)

Table 3 shows cases and treatment characteristics by center type, CACON, and others. The proportion of subtypes was similar, with a predominance of STS, followed by BS and GIST, independent of the center type. CACON had a lower rate of sarcoma not otherwise specified (13.6%) when compared with other centers (18.5%). The proportion of patients starting their treatment within 60 days of diagnosis at CACON was 59.3% and 62.3% at other centers. Metastatic cases at diagnosis were 32.9% at CACON and 36.9% at others. The number of cases without previous diagnosis or treatment was 45.3% at CACON and 43.3% at others. The proportion of cases being treated at the same institution from the beginning was similar at CACON and at other centers (69% and 71.9%, respectively). In both hospital types, surgery was the most common first treatment followed by chemotherapy and radiotherapy.

**TABLE 3:**
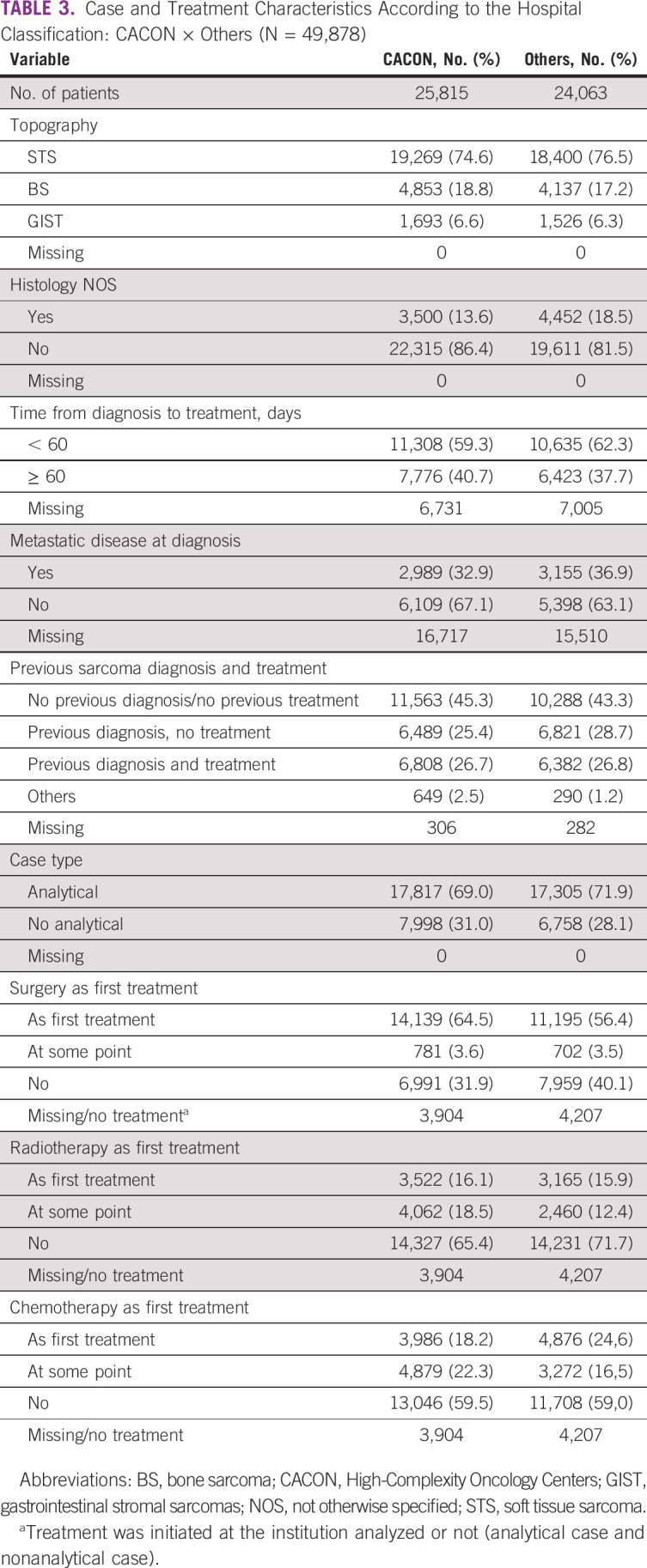
Case and Treatment Characteristics According to the Hospital Classification: CACON × Others (N = 49,878)

Per Table 4, analysis of geographical area and patient volume distribution according to the hospital classification, a greatest number of hospitals were not classified as high-complexity cancer centers. Ten hospitals achieved the established threshold for high-volume center (> 70 patients/year for 3 consecutive years), of which seven were CACON. The SE region contains the highest number of CACONs (52%).

**TABLE 4:**
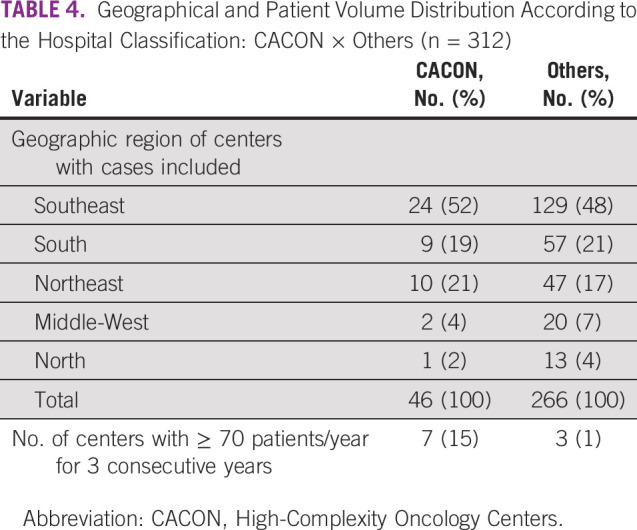
Geographical and Patient Volume Distribution According to the Hospital Classification: CACON × Others (n = 312)

## DISCUSSION

This study presents demographic data of patients diagnosed with sarcoma and describes their main diagnosis and treatment pathways in Brazil, a topic poorly investigated nationally. As a retrospective study using a hospital-based pool of data, our analysis has some limitations. First, the cohort does not represent the entire Brazilian population. Second, data collection is not uniform since it is mainly used to evaluate the metrics of care at each center. Although the proportion of missing data has not varied over time, the retrospective design collecting data of 312 centers may justify the lack of information about some variables. In addition, during the years analyzed, many changes in sarcoma classification and staging have taken place. Also, the loss of cases or misdiagnosis is a real possibility since the histologic inclusion criteria used, the ICD-O3,^19^ do not encompass many of the more recent sarcomas included in the WHO classification.^4^ Considering these limitations, some interesting data could be evaluated.

Despite the population heterogeneity across the country, subtype distribution was consistent with population-based data reports, showing a soft tissue predominance followed by bone and GIST in all regions.^20^ It correlates with the fact that the origin of most sarcomas is unknown. Risk factors for STS include age (about one third is 65 years and older), previous radiation treatment, previous cancers, and rare family genetic conditions.^7^ Incidence rates could not be calculated when the hospital-based registry did not give precise information about the population covered and health migration data are unknown.

Cases were concentrated in the SE region, the most populous, where the majority of high-complexity centers are also located. However, the classification as a high-complexity cancer center does not imply good sarcoma care. In our analysis, CACONs and others had equivalent proportions of pathologic diagnosis as sarcoma NOS, metastatic cases at diagnosis, and similar sequencing of treatments. For a center to be considered as a sarcoma specialist unit, a multidisciplinary team of experts, clinical practice guidelines adhesion, adequate facilities, and the patients/volume per year are crucial items.^7^ Given the lack of recognized sarcoma reference centers in Brazil, we focused on analyzing the number of new cases/year in each hospital. There is no consensus about the minimum volume for a center to be considered a reference for sarcoma care. Different thresholds are used, varying from 70 to 100, depending on the country and population size.^7,12^ Assuming the volume threshold of at least 70 patients/year per three consecutive years, only seven CACONs could be considered a specialist center.

Another important variable to evaluate quality of care is the time from diagnosis to the start of treatment. In Brazil, by national law (12.732/12—May 23, 2013), cancer treatment must be implemented within 60 days of diagnosis and should be carried out mostly in CACONs. But in fact, there was no major difference in timing between CACON and other centers, with as many as 40% of patients with sarcomas starting treatment after 60 days of diagnosis. These data may reflect the difficulties of directing a patient with sarcoma to an appropriate center and the absence of formal strategies to treat the disease. Treatment delay and advanced stage at diagnosis are indicators of poor prognosis. A number of obstacles, such as access barriers, the absence of a specific flow for those patients, and inadequate resources, may exacerbate the negative effect of such indicators. An additional concern is the lack of formal subspecialization in mesenchymal tumors in Brazil. Some patients are treated by general oncologists or general surgeons and orthopedists, who may not be adequately trained in sarcoma management.

Tumor size and metastatic spreading at diagnosis are also important prognostic factors in sarcomas.^21^ In the current analysis, the rate of metastatic disease was around 30% at diagnosis, which is in accordance with other records,^11,19,22^ and did not differ considerably according to the region or center type. The rate of locally advanced disease could not be calculated. Despite the high missing data rate in all regions, the proportion over the years analyzed was stable. The number of sarcomas diagnosed increased over time. Improvement in the quality of registries, as a result of population growth in Brazil, and also better integration among different data systems can be responsible for the growth in cases. It is also important to note that sarcoma diagnosis is becoming more accurate because of better diagnostic techniques and well-trained personnel, avoiding many erroneous results. On the other hand, the similarities across regions and centers may reinforce the absence of a specific flow for patients with sarcoma to enter the cancer care pathway. Awareness from caregivers at the basic level is fundamental to identifying the disease in its early stages and referring the patients in a timely manner.^23^

Proper treatment is also challenging, owing to rarity and the need for expertise to conduct decisions. The present data reveal that most patients have only surgery as upfront treatment. Radiotherapy and chemotherapy were poorly used as ancillary methods. This may reflect the absence of multidisciplinary discussions culminating in upfront surgery, even without diagnostic biopsy and the difficulties in referring those cases to perioperative chemotherapy and radiotherapy in high-complexity centers. The continuity of treatment was not evaluated because of insufficient data.

There is much evidence to support early recognition and referral to a specialized center or network as factors that improve outcomes in patients with sarcoma.^8-10,20,21,24,25^ This is due to a multidisciplinary approach, given by teams used to treat a high number of cases annually. Conversely, countries without policies to uniformly approach the disease have lower 5-year survival rates.^1^ In such a large country as Brazil, the hub and spoke model could be an effective solution to shorten the distances and to improve care. This model arranges service delivery assets into a network consisting of an anchor establishment (hub), which offers a full set of services, complemented by secondary establishments (spokes), which offer more limited service arrays, routing patients needing more intensive services to the hub for treatment. If properly organized, it allows access to a fluid and efficient care pathway close to home and affords many benefits for health care providers.

A well-connected network of experienced professionals could also avoid incorrect diagnoses, which is clearly a cornerstone for those patients and for registry. There are no data about pathology peer review for sarcomas in Brazil. Actually, this is not a common practice. In our analysis, almost 15% of the cases were classified as undifferentiated or unclassified sarcomas, pointing to the need for more diagnostic tools and expertise to better classify these tumors and offer treatment accordingly.

Initial care improvement is not just a matter for specialized oncology professionals. General doctors, especially surgeons, must be aware of when suspecting a sarcoma and refer immediately those patients to a proper center or discuss the initial approach within a well-connected network.^7,25^ To address this issue, awareness campaigns may help to spread information in the community and avoid delayed diagnoses and inadequate treatments. Recently, a Brazilian national law (13.896/19—April 28, 2020) was implemented to guarantee that, if cancer is suspected, tests to confirm the diagnosis should be performed within 30 days. But to set a single deadline for all cancer types may be inappropriate given the peculiarities in diagnosis, the sequencing for the examinations required, and the evolution of each histology. Initiatives that strengthen primary care and are histology-driven could achieve the desired result, which is to shorten the time from suspicion to adequate diagnosis.

A recent effort called SELNET project (Sarcoma European Latin American Network)^26^ was implemented, aiming to meliorate diagnosis and clinical care in sarcomas in Latin America. The core of the work focuses on improving diagnosis and prognosis of patients with adult sarcoma through the creation of pathologic diagnosis networks, multidisciplinary boards, and international registry–based and intercontinental sarcoma biobanks to promote clinical and translational research. It is an international initiative that needs a national fertile ground to blossom.

The difficulties reported reflect the need for better mapping and shaping sarcoma patient pathways. High-quality national population–based cancer registries are fundamental to address the disease reality and the barriers during the clinical care experience.

In conclusion, this article highlights the need for further research on the profile of patients with sarcoma in Brazil and the importance of providing them a more effective diagnostic and therapeutic approach. This initiative is critical not just for planning treatment strategies but also for allocating medical resources. In a continental country such as Brazil, the hub and spoke system could be an efficient manner of spreading high-quality care.

Nevertheless, further studies are required to clarify the sarcoma burden not only in Brazil but worldwide; but, for this, networking is crucial. Particularly, the rarity imposes more limitations on the analysis, given the possibility of greater difficulty in diagnosis and, therefore, error in the registration as a consequence of the original diagnostic mistake. The connection with clinical societies may increase the awareness of improving data quality in the cancer registry, especially in rare cancers. This effort is necessary to improve quality of care and patient outcomes.
